# AMP‐activated protein kinase regulates cancer cell growth and metabolism via nuclear and mitochondria events

**DOI:** 10.1111/jcmm.14279

**Published:** 2019-04-16

**Authors:** Shanshan Jiang, Yan Wang, Lingyu Luo, Fuli Shi, Junrong Zou, Hui Lin, Ying Ying, Yunfei Luo, Zhan Zhan, Peijun Liu, Bo Zhu, Deqiang Huang, Zhijun Luo

**Affiliations:** ^1^ Institute of Digestive Diseases The First Affiliated Hospital Nanchang China; ^2^ Jiangxi Provincial Key Laboratory of Tumor Pathogens and Molecular Pathology Department of Pathophysiology Schools of Basic Sciences Nanchang China; ^3^ Institute of Hematological Research Shaanxi Provincial People's Hospital Xi'an China; ^4^ Pharmaceutical Sciences Nanchang University Jiangxi Medical College Nanchang China; ^5^ Center for Translational Medicine The First Affiliated Hospital of Xi'an Jiaotong University Xi'an China; ^6^ Department of Pharmacology and Experimental Therapeutics Boston University School of Medicine Boston Massachusetts

**Keywords:** AMP‐activated protein kinase, cancer growth, glycolysis, mitochondrial function, oxidative phosphorylation, Warburg effect

## Abstract

Adenine monophosphate‐activated protein kinase (AMPK) is a fuel sensing enzyme that is activated in shortage of energy and inhibited in its surplus. Cancer is a metabolic disease characteristic of aerobic glycolysis, namely Warburg effect, and possesses heterogeneity featured by spatiotemporal hypoxia and normoxia, where AMPK is deeply implicated. The present study delineates the regulation of mitochondrial functions by AMPK in cancer cells. On the one hand, AMPKα subunit binds to mitochondria independently of β subunit and targeting AMPK to mitochondria facilitates oxidative phosphorylation and fatty acid oxidation, and inhibits glycolysis. As such, mitochondrial AMPK inhibits the growth of cancer cells and tumorigenesis. On the other hand, ablation of the β subunits completely abolishes AMPK activity and simultaneously leads to decreases in mitochondria DNA and protein contents. The effect of the β deletion is rescued by overexpression of the active mutant of bulky AMPKα1 subunit. In conjunction, the transcriptional factors PGC1α and Nrf‐1 are up‐regulated by LKB1/AMPK, an event that is abolished in the absence of the β subunits. Intriguingly, the stimulation of mitochondria biogenesis is not achieved by mitochondria‐targeted AMPK. Therefore, our study suggests that AMPK inhibits cancer cell growth and tumorigenesis via regulation of mitochondria‐mediated metabolism.

## 1 INTRODUCTION

Cancer arises from mutations of a series of genes regulating cell growth including tumour promoters and tumour suppressors. To suffice the demands of rapid proliferation, transformed cells must generate sufficient energy to fuel the increased anabolic program. Indeed, the energy generating mechanism in cancer cells is different from normal cells, which was first described by Warburg in early 1920s, also called Warburg effect.^1^ Namely, cancer cells utilize glycolysis as the major energy source even under aerobic conditions, while normal cells mainly entail mitochondrial oxidative phosphorylation.

Although the generation of ATP through glycolysis is less efficient, the glycolytic rate in cancer cells can be up to 200 times higher than normal cells even in the presence of oxygen, assuring immediate availability of ATP for anabolic demands of cancer cells.^7^ The adaptation of cancer cells to glycolysis appears to have several advantages.^2^ First, it is beneficial to accelerated growth. The cancer cells are adapted to acidic microenvironment resulting from release of lactic acid, the end‐product of glycolysis, which is beneficial to activation of matrix metalloproteinases, enzymes for cleavages of extracellular matrix and promotion of cell migration and invasion.^3^ Second, glycolysis provides cancer cells with various metabolic precursors that are used for the synthesis of amino acids, nucleotides and lipids, as well as reducing power.^4^ Third, many oncogenes and tumour suppressors, previously known to control cell growth, have now emerged as modulators of glycolysis.^5^ For example, the *TP53* tumour suppressor, the most frequently mutated gene in human cancer, supports oxygen‐dependent energy production by promoting mitochondrial biogenesis and suppresses glycolysis.^6^ Critical components in the glycolytic pathway can be directly or indirectly regulated at all levels by oncogenic or tumour suppressive proteins. For instances, hypoxic inducing factor 1α (HIF1α), a master regulator for glycolysis, is activated under hypoxic condition and destructed on normoxia by proteosomal degradation. However, HIF1α is activated by many oncogenic proteins and thus contributes to aerobic glycolysis.^5^

Adenine monophosphate‐activated protein kinase (AMPK) is highly conserved in eukaryotes and acts as a fuel gauge that senses energy crisis. In mammalian cells, AMPK consists of three subunits, catalytic α (α1, α2) subunits and regulatory β (β1, β2) and γ (γ1, γ2, γ3) subunits.^8^ When AMP level or AMP to ATP ratio is increased, AMPK activation is initiated by binding of AMP to the γ subunit, followed with phosphorylation of T172 in the activation loop of the α subunit by the liver kinase B1 (LKB1).^9^, ^10^ Likewise, pharmacological agents that mimic calorie restriction to increase cellular AMP can activate AMPK, which include 5‐amino‐4‐imidazolecarboxamide riboside‐1‐β‐d‐ribofuranoside(AICAR), metformin, berberine, etc. Salicylate and A‐769662 directly bind to the interface between α and β subunits and prevent dephosphorylation. In addition, T172 can be phosphorylated by other kinases, such as calmodulin‐dependent protein kinase β (CaMKKβ),^11^, ^12^ TGF‐β activated kinase 1 (TAK1),^13^, ^14^ and possibly mixed lineage kinase 3 (MLK3).^15^ Another mechanism of AMPK activation was recently identified in lysosomes sensing the absence of fructose‐1,6‐biphosphate (FBP), independent of AMP.^16^ In the presence of glucose, FBP binds to aldose in the outer membrane of lysosome, while in the absence of FBP owing to lack of glucose, aldose dissociates from lysosome and is replaced by LKB1 to phosphorylate AMPK in situ. Upon activation, AMPK phosphorylates a broad array of substrates. As AMPK is suppressed by high glucose and lipids, and activated by metformin, the first line drug for type 2 diabetes, it is a well‐received therapeutic target for metabolic disorders.^17^ In fact, the activation of AMPK by pharmacological agents or exercise can alleviate hyperglycaemia and hyperlipidemia and enhance insulin sensitivity.^18^, ^19^, ^20^ More than this, recent studies have depicted the roles of AMPK in various other functions such as fibrosis, epithelial to mesenchymal transition (EMT), cancer growth and progression.^17^, ^21^, ^22^, ^23^

With regard to the role of AMPK in tumorigenesis, opposite views have been put forward. While a great number of studies have shown that AMPK plays inhibitory roles in the growth of cancer cells and tumorigenesis, many studies have illustrated the tumour‐protecting and promoting effects of AMPK, which are correlated to its ability to respond to stress and confer protection to cancer cells.^24^ The present study attempts to delineate the regulatory role of AMPK in energy metabolism and cancer cell growth by focusing on regulation of mitochondria. To this end, we investigated whether bulky cellular or mitochondrial AMPK regulated mitochondria biogenesis, glycolysis and oxidative phosphorylation. We also assessed if these functions of AMPK correlate to its ability to impact on the growth of cancer cells and tumorigenesis.

## 2 MATERIALS AND METHODS

### 2.1 Materials and reagents

Metformin, AICAR, doxycycline, berberine and mouse monoclonal antibody against flag were purchased from Sigma Aldrich (St. Louis, MO, USA). A769962 was from Bio‐Techne Corporation (Minneapolis, MN, USA). Mitochondria isolate kit (MitoSciences) was from Abcam (Cambridge, MA, USA). The following antibodies were purchased from Cell Signaling Technology (Danvers, MA, USA): β‐actin, AMPKα total and phospho‐T172, AMPKβ, and phospho‐ACC(S79). 3‐(4,5‐dimethylthiazol‐2‐yl)‐2,5‐diphenyltetrazolium bromide (MTT), Lipofectamine2000, mouse monoclonal antibody against mitochondrial ATP synthase β subunit, Cyanine‐3‐conjugated goat anti‐rabbit antibody and FITC‐conjugated goat antimouse antibody were from Life Technologies (Grand Island, NY, USA). Mouse monoclonal antibody against LKB1 was from Santa Cruz Technology (Santa Cruz, CA, USA). Gibson Assembly Cloning kit was purchased from New England Biolabs (Ipswich, MA, USA). mRNA and DNA extraction kits were from Qiagen (Germantown, MD, USA). Seahorse XF Glycolysis Stress Test Kit and Seahorse XF Cell Mito Stress Test Kit were purchased from Agilent (Santa Clara, CA, USA).

### 2.2 Cell culture and transfection

The lung adenocarcinoma A549 cell line and A549‐LKB1 cell line were established previously^26^ and the HEK293 cell line was purchased from ATCC. All these three cell lines were cultured in Dulbecco's modified Eagle's medium (DMEM; Hyclone), supplemented with 10% foetal bovine serum (Hyclone) and 5% 100 IU/mL penicillin‐100 μg/mL streptomycin at 37°C and 5% CO_2_. Transfection was performed with Lipofectamine2000 according to the protocol provided by the manufacturer.

### 2.3 Construction of expression vector of AMPKα1 variants and guide RNA plasmid of AMPK β subunits

cDNAs encoding variants of AMPKα1 tagged with the flag epitope were subcloned to pCDNA3.1(‐) vector and transfected into HEK293T cells.

To carry out gene editing of β1 and β2 subunits of AMPK, Cas9GFP and pX330 were obtained from Addgene (Cambridge, MA, USA). The plasmid for guide RNA was reconstructed based on pCDNA3.1(‐) as follows: (a) replace neomycin gene with puromycin gene, (b) delete CMV promoter and transcriptional termination sequence on pCDNA3.1 with NruI and BbsI, (c) amplify human U6 promoter and terminator by PCR, and ligate the two pieces of DNA with the Gibson Assembly Cloning kit. Oligonucleotides (Table 1) for the guide RNA were subcloned at BbsI site.

**Table 1:** Primers synthesized for RT‐PCR analysis

Gene name	Forward primer	Reverse primer
β1 subunits guide RNA‐1	5′CACCT_471_GGCCTGGCAGCATGATC_488_ starting from W_61_	5′AAACGATCATGCTGCCAGGCCA
β1 subunits guide RNA‐2	5′CACCA_598_AGTTTACTCCAGTTGTTGA_579_ staring from ~K_96_	5′AAACTCAACAACTGGAGTAAACTT
β2 subunits guide RNA‐1	5′CACCA_355_GAGTTTGTATCATGGCAGC_374_ starting from ~E57	5′AAACGCTGCCATGATACAAACTCT
β2 subunits guide RNA‐2	5′CACC_449_CTTGCCTCCTTCAGACCAGC_429_ start from ~D_81_	5′AAACGCTGGTCTGAAGGAGGCAAG
tRNA‐Leu(UUR)	5′CACCCAAGAACAGGGTTTGT	5′TGGCCATGGGTATGTTGTTA
β2‐microglobulin	5′TGCTGTCTCCATGTTTGATGTATCT	5′TCTCTGCTCCCCACCTCTAAGT
Nrf‐1	5′TCGCAAGTGGATCCTGACTG	5′GGTGACTGCGCTGTCTGATA
PGC1α	5′ACCACAAACGATGACCCTCC	5′GCCTGCAGTTCCAGAGAGTT

A pool of four guide RNA plasmids was cotransfected with Cas9GFP into A549 cells and 2 days after transfection, the cells were selected with puromycin, which was then removed after 3 days. The clones were amplified for 14 days and isolated. Knockout was identified by Western blot.

### 2.4 Preparation of virus expressing AMPK

cDNA encoding the active mutant of AMPK was engineered by tagging mitochondrial binding sequence from cytochrome C oxidase and a flag epitope at its aminoterminus. The chimeric cDNA was subcloned into a lentiviral expression vector under the control of Tet‐off promoter, the lentivirus was prepared, and cells were infected as previously described.^26^

Adenovirus expressing the active mutant of AMPK tagged only with the flag epitope was prepared as described previously.^37^

### 2.5 Mitochondria fractionation

Mitochondria fraction was prepared using a kit (MitoSciences) provided by Abcam according to the protocol provided.

To prepare mitochondria from rat, the liver was minced into small piece and about 0.6 mg was homogenized in 6 mL of buffer A at 4°C. The following steps are the same as for the cultured cells.

### 2.6 Western blot

Western blot was performed as we described previously.^37^

### 2.7 MTT assay

The assay was performed according to standard protocol.^37^

### 2.8 Wound healing

The assay was performed as described previously.^37^

### 2.9 Clonogenic assay

The assay was conducted according to the protocol described by Franken et al.^51^ When the colonies were visible, the plates were washed with PBS, fixed with fixation solution (acetic acid/methanol, 1:7 v/v), stained with crystal violet (0.5%), and washed twice again. Photographs were taken.

### 2.10 Measurement of mitochondrial DNA to genomic DNA ratio

The assay was performed according to Rooney et al.^52^ Cellular DNA was extracted using the DNA extraction kit (Qiagen). Mitochondrial DNA and genomic DNA were evaluated by qPCR using the primers (Table 1). The relative ratio of mitochondrial DNA to genomic DNA was assessed using the equation: 2 × 2^∆CT^. ∆CT = (nucDNA)CT − (mitoDNA)CT.

### 2.11 Quantitative PCR

The expression of mRNA was quantified by qPCR with the ABI Step One Plus PCR System using SYBRGREEN PCR Master Mix 2× reagent in 20 μL reaction volume according to protocol provided by manufacture (Applied Biosystems, Foster City, CA, USA). The primer sequence is listed in Table 1. Each sample was amplified in triplicates and normalized with Glyceraldehyde 3‐phosphate dehydrogenase (GAPDH) expression level. Results were evaluated by the comparative threshold cycle value method (2^−ΔΔCt^) for relative quantification of gene expression.^26^

### 2.12 Glycolysis and oxygen consumption assays

Both assays were performed with Seahorse Bioscience XF Analyzer. Glycolysis rate was measured using Seahorse XF Glycolysis Stress Test Kit and oxygen consumption rate (OCR) using Seahorse XF Cell Mito Stress Test Kit.

### 2.13 Tumour animal model

Animal protocol was approved by institutional animal care and use committee. For Xenograft model, A549 cells (10^7^) were resuspended in 100 μL phenol red‐free DMEM medium and mixed with 100 μL matrigel (Corning, Tewksbury, MA, USA) for each injection. The cell suspensions were then injected subcutaneously into the back of nude mice (5 weeks old males) (Purchased from Nanjing University Biomedicine Institute, Nanjing, China). Each group consisted of 6 mice. Ten days after inoculation, tumours were measured with callipers every 3 days and tumour volume was calculated using the formula W2_L/2 (W, shorter diameter; L, longer diameter) (mm^3^). Animals were killed when tumour diameter reached 1.5 cm, which takes approximately 39 days after inoculation, and tumour removed.

For allograft model, B16F10 cells (5 × 10^6^) were injected subcutaneously into the back of C57 mice (Purchased from Nanjing University Biomedicine Institute, Nanjing, China) (6 each group) using similar method also described above except without matrigel. One week after injection, animals were gavaged with 0.1 mL suspension of berberine in PBS (27 mg/mL, 135 mg/kg) every other day. The tumour sizes were measured every 3 days.

### 2.14 Immunofluorescence

Cells were fixed in 4% paraformaldehyde in PBS and then washed with PBS. For AMPK β subunit, samples were blocked with 10% normal goat serum (NGS) for 30 minutes, and incubated with rabbit monoclonal antibody for 2 hours. After washing three times with PBS, the samples were incubated with Cyanine‐3‐conjugated goat anti‐rabbit antibody (Life Technologies) for 1 hour. For ATP synthase β subunit, the second antibody was FITC‐conjugated goat antimouse IgG. All antibodies were diluted in PBS containing 2% NGS. After immunofluorescent staining, the cells were also counterstained with DAPI (0.5 μg/mL for 5 minutes) to detect the nuclei and the coverslips mounted in spectrometric grade glycerol and sealed with nail polish. Fluorescent images were taken under confocal microscope.

### 2.15 Malonyl CoA assay

Malonyl CoA was radioisotopically assayed as described previously.^53^

### 2.16 Statistical analysis

Significance of differences amongst groups was determined by two‐tailed Student's *t* test. Statistical analysis of the mean differences between groups that have been split on two independent variables (eg volume × time) were examined with GraphPad InStat by using two‐way ANOVA. *P* < 0.05 was set for significance.

## 3 RESULTS

### 3.1 Localization of AMPK in mitochondria

**Figure 1:**
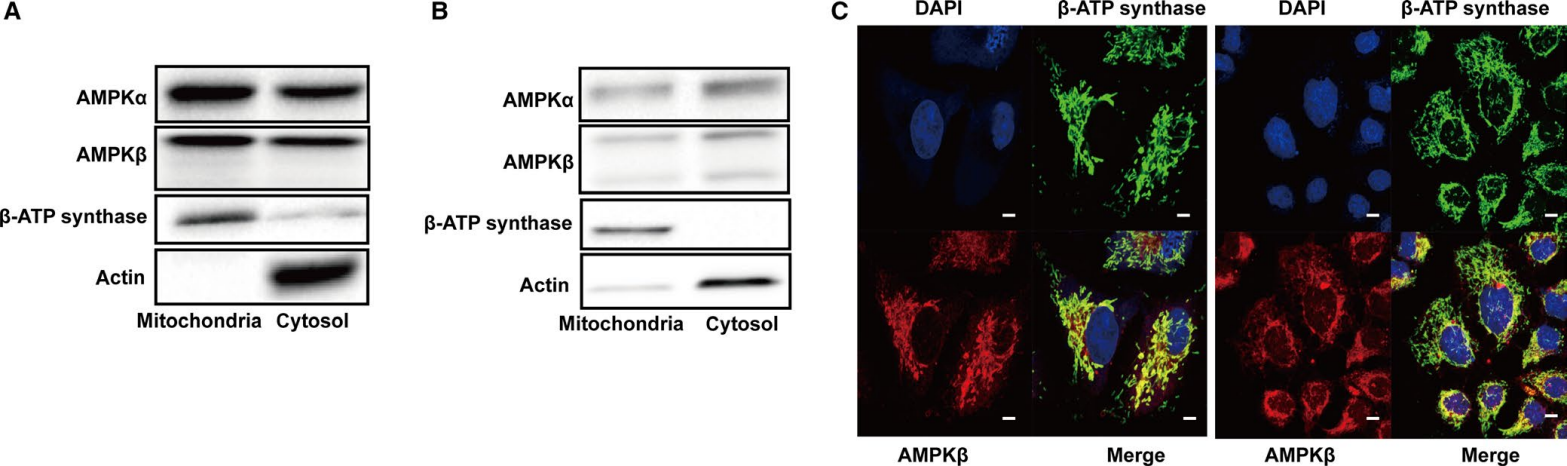
Localization of AMPK in mitochondria. Mitochondria and cytosol fractions were prepared from rat liver (A) and A549 cells (B) and blotted with antibodies as indicated. C, HEK293 and A549 cells were immunofluorescently labelled with anti‐AMPKβ subunit (red), ATP synthase β subunit (green), and DAPI. Images were taken under confocal microscope. Bar scale: 50 μm

To examine if AMPK is localized to mitochondria, we first performed biochemical fractionation of cell extracts from rat liver and A549 cells. As shown in Figure 1A and B, both AMPKα and β subunits were found in the mitochondrial fraction. Since biochemical mitochondrial fractionation might be contaminated by other cellular compartments, we then conducted immunofluorescent staining of AMPKβ and mitochondrial ATP synthase β subunit (Figure 1C). In this experiment, we labelled both HEK293 cells and A549 cells with anti‐β subunit antibody (red) and subsequently with anti‐ATP synthase β subunit antibody (green). The results revealed considerable amount of superimposed signals as discerned by orange colour although they did not completely overlap, as expected. All these results indicate that part of the AMPK holoenzyme is localized to mitochondria.

**Figure 2:**
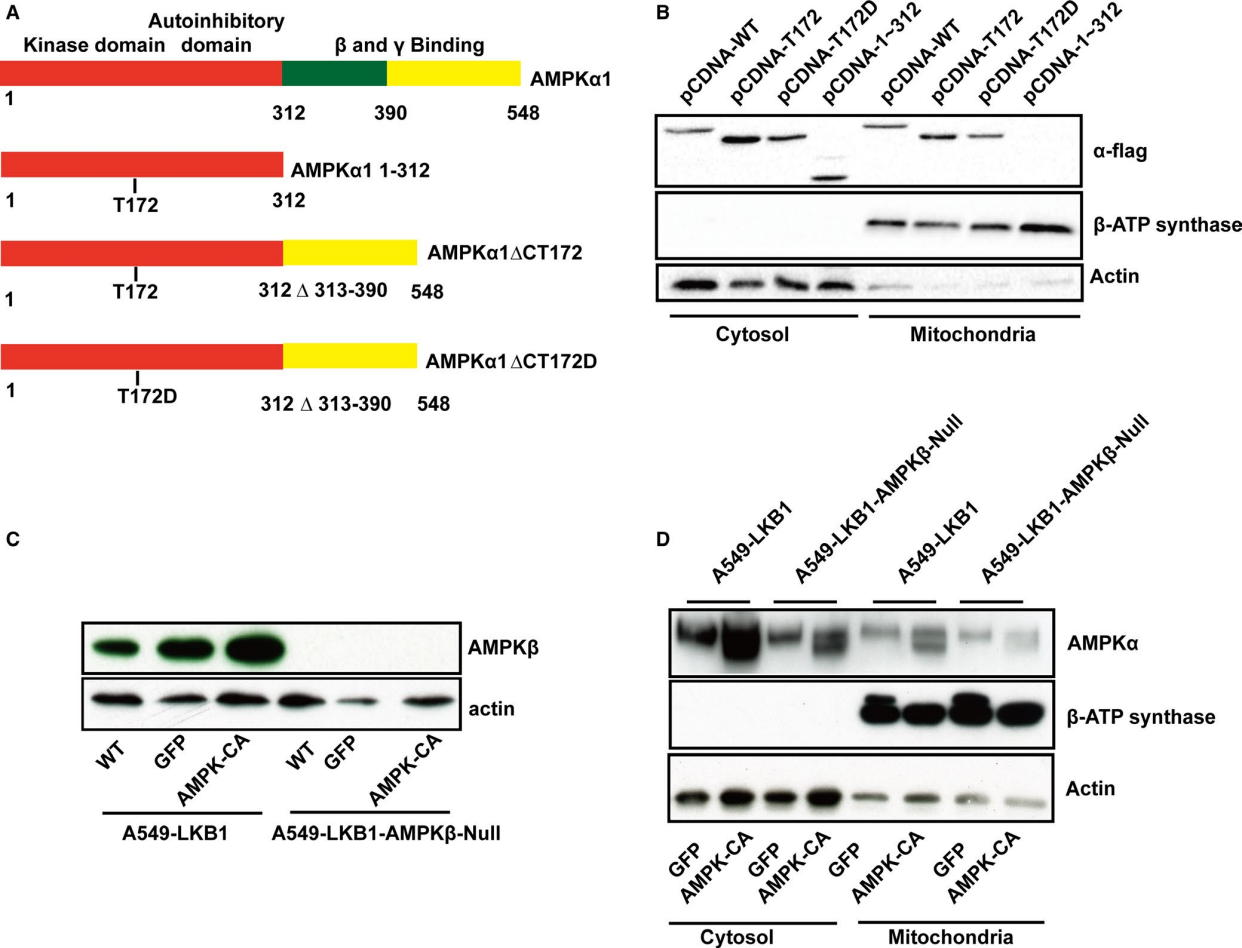
Mitochondria binding domain of AMPKα1. A, Schematic diagram of deletion mutations of AMPKα1 subunits. B, cDNAs encoding a variants were expressed in HEK293 cells and cell extracts blotted with antibodies as indicated. C, β1 and β2 subunits were deleted in A549‐LKB1 cells (A549‐LKB1‐AMPKβ null) and the cells infected with adenovirus expressing GFP or the active mutant of AMPK (α1ΔC‐T172D) (labelled as AMPK‐CA). Western blot was performed with antibodies as indicated. D, Mitochondrial and cytosol fractions were prepared from the cells as described in C and blotted with antibodies as indicated

Next, we determined the binding domain on AMPK. To this end, we constructed a series of mutants of AMPKα1, prepared mitochondria fraction after transient transfection of them into HEK293 cells and performed Western blot. Our results revealed that the truncated kinase domain (aa1‐312) was unable to bind mitochondria and the binding domain was comprised within the carboxyl moiety within aa391 to 548 (Figure 2B). Since this domain contains a binding site for β subunit, we ascertained if the association is mediated through β subunit. Thus, we employed Crispr/Cas9 gene editing technology to knock out both β1 and β2 subunits in A549 cells expressing LKB1 (A549‐LKB1) (Figure 2C). Using this cell line, we infected adenovirus expressing the active mutant of AMPK or GFP virus as a control, and then performed mitochondria fractionation to determine the distribution of AMPK. Figure 2D shows that both endogenous α and recombinant α1 subunits associated with mitochondria regardless that the β subunits were absent (Figure 2C).

### 3.2 Targeting AMPK to mitochondria inhibits tumorigenesis

**Figure 3:**
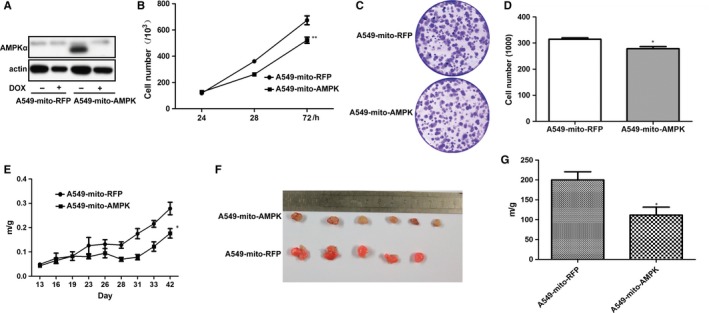
Biological functions of mitochondria‐targeted active mutant of α1 subunit. A, Inducible expression of mitochondria‐targeted expression of α1 subunit. A549 cells expressing mitochondria‐targeted RFP or the active mutant of AMPKα1 were treated with doxycycline (2 μg/mL) for 2 d and cell extracts blotted with antibodies as indicated. B, Growth curve of the cells expressing the mitochondria‐targeted active mutant of α1 or RFP. Cells were plated on 6 well plates and counted at 24, 48 and 72 h. Averages of cell counts (±SD, n = 3) were plotted. Statistical analysis was performed with two‐way ANOVA. **P < 0.01. C, Clonogenic assays. Cells were plated and 2 wk later plates stained and images taken. D, Clones from D were counted in randomly selected areas after photography. Averages (±SD, n = 4) were plotted. Student's *t* test was used to test significance. *P < 0.05. E‐G, Cells expressing RFP or active mito‐AMPK were injected subcutaneously into back of nude mice (six for each cell lines, five developed from RFP cells). Tumour growth were monitored and volumes calculated (E). At the end of experiments, tumours were removed (F) and weighed (G). Statistical analysis for tumour volumes was performed with two‐way ANOVA and *t* test for tumour weight. *P < 0.05

Previous studies have shown that AMPK suppresses Warburg effect, so as to inhibit lymphomagenesis.^25^ To test if binding of AMPK to mitochondria leads to an inhibition of tumorigenesis, we engineered a construct encoding the active mutant of AMPKα1 in which a mitochondrial targeting site from cytochrome C oxidase and flag epitope were subsequently tagged to its aminoterminus. This chimeric cDNA was then inserted into a lentiviral vector under the control of Tet‐off system. We made the stable cell line of A549 cells and demonstrated that the expression of α1 could be successfully shut off by doxycycline (Figure 3A). As doxycycline is known to inhibit mitochondria function, we used mitochondria‐targeted RFP as a control in most of experiments in this work. We then measured the growth profile of the cells expressing the active α1 mutant. Our results showed that the mitochondria‐targeted active AMPKα1 remarkably inhibited the growth of the cells and their ability to form colonies, as compared to RFP control (Figure 3B‐D) (*P* < 0.05). In an attempt to test if the effect of mitochondria‐targeted AMPK α1 is due to sequestering factors for cell growth to mitochondria, we prepared mitochondria‐targeted AMPK wild‐type and kinase‐dead mutant and expressed them in A549 cells. Our results did not suggest this was the mechanism, but rather the kinase activity is required (data not shown).

We conducted subcutaneous injection of these two cells into nude mice and observed the tumour growth curve. The results showed that the cells with the mitochondria‐targeted AMPK mutant developed tumour slower and their average tumour weight was also less than the control cells (*P* < 0.05) (Figure 3E‐G). Altogether, our data demonstrate that binding of AMPK to mitochondria suppresses the growth of cancer cells, suggesting that it occurs via inhibition of the Warburg effect.

### 3.3 The effect of AMPKβ subunits knockout on mitochondria biogenesis

**Figure 4:**
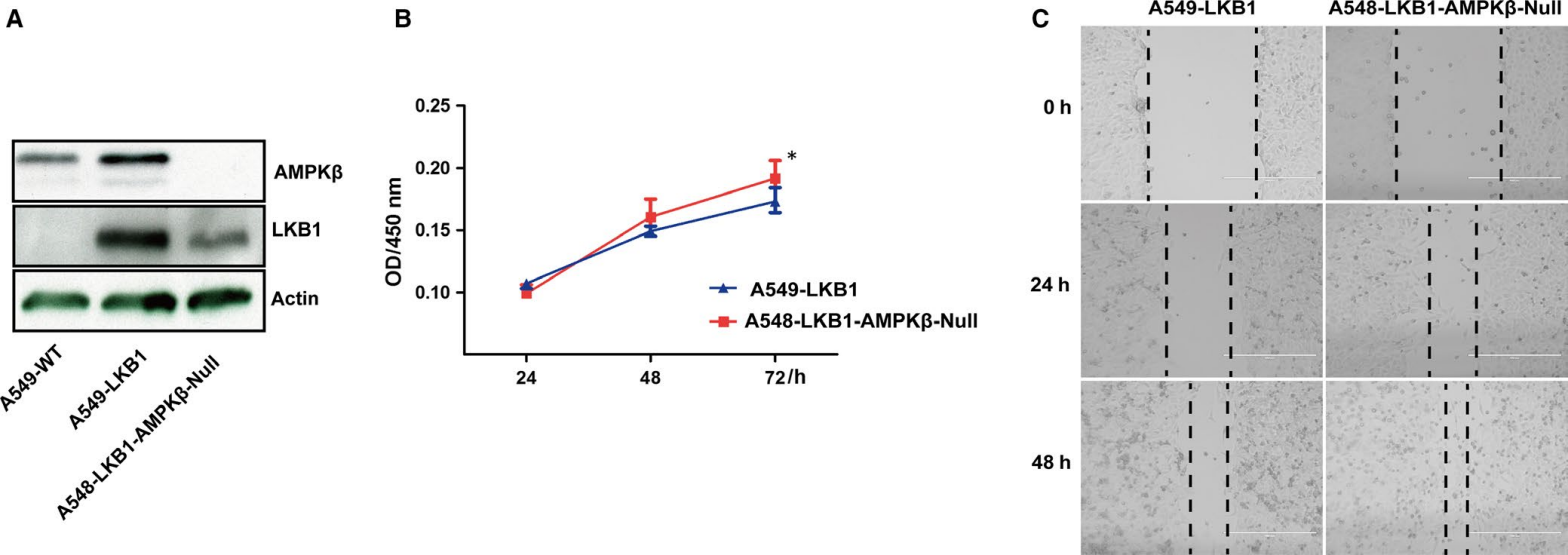
Deletion of β subunits affects cell behaviour. A, Western blot analysis of A549 cells and A549 LKB1 cells with or without β subunits deleted. B, Cell viability performed with MTT assays. Statistical analysis was performed as for Figure 3C. *P < 0.05. C, Wound healing. Assays were performed as described in Section 2

A549 cells contained a loss‐of‐functional mutation of LKB1. We have found that the growth of A549‐LKB1 was compromised and the cells failed to develop tumour in nude mice.^26^ To assess if the inhibitory effect of LKB1 on the cancer cell growth is mediated by AMPK, we deleted β1 and β2 subunits on the context of A549‐LKB1 (Figures 2C and 4A). Our results showed that cell viability and wound healing were increased after ablation of the β subunits, suggesting that the inhibitory effects of LKB1 on the growth and cell migration were mediated by AMPK (Figure 4B and C).

**Figure 5:**
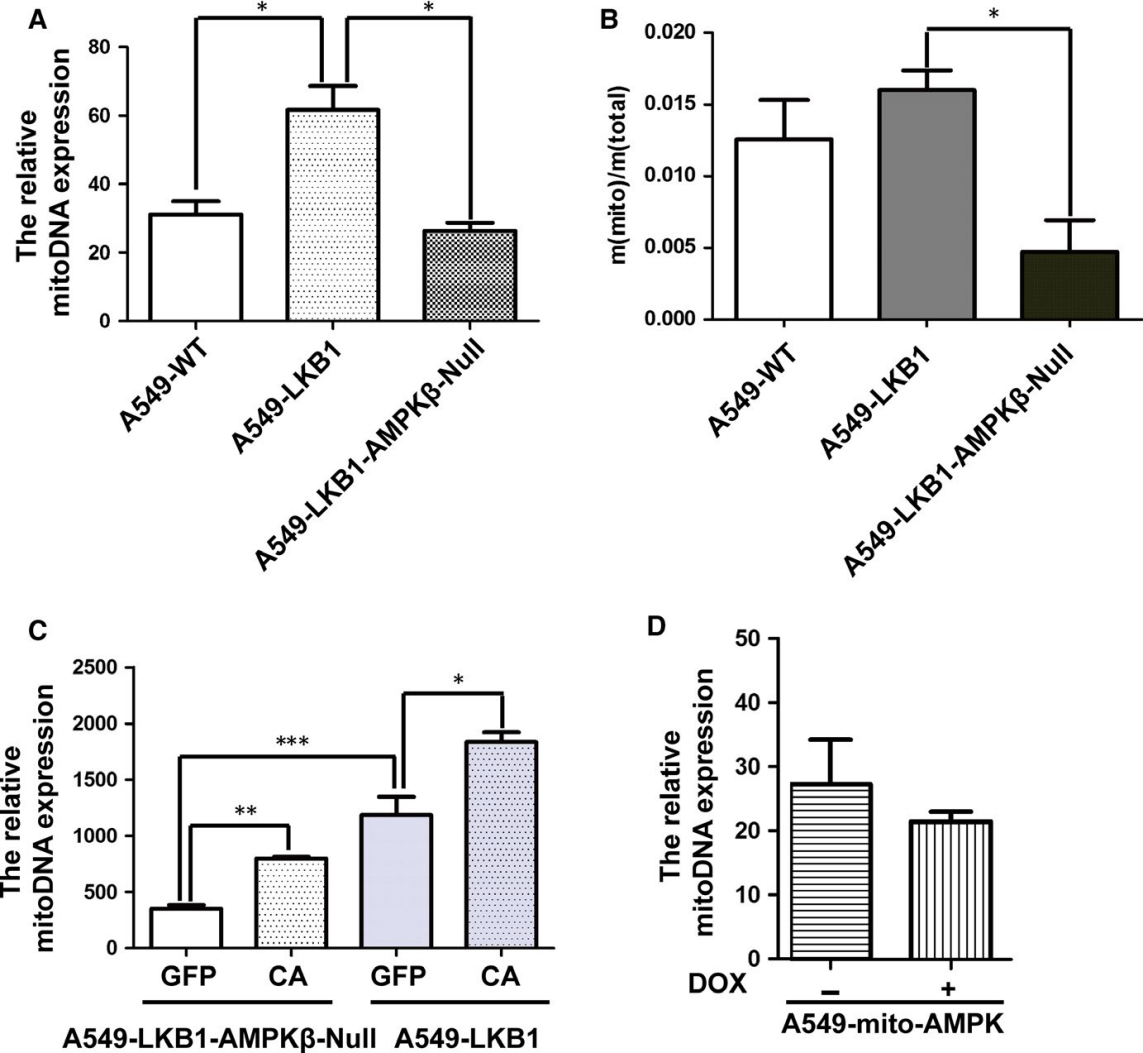
The effect of AMPK on mitochondria content. A, Ratio of mitochondrial DNA to genomic DNA was determined using qPCR from cell lines as indicated. B, Ratio of mitochondrial protein to total proteins was determined by protein assay. C, Ratio of mitochondria DNA to genomic DNA in the A549‐LKB1 cells with or without β subunits knocked out after infection of adenovirus expressing GFP or active α1 subunit. D, Ratio of mitochondria DNA to genomic DNA in A549 cells expressing mitochondria‐targeted active AMPK mutant under the control of Tet‐off system (the cells were treated with doxycycline [2 μg/mL] for 2 d). Student's *t* test was used to test significance. *P < 0.05, **P < 0.01, ***P < 0.001

We postulated that AMPK counteracted the Warburg effect, leading to the inhibition of the cancer cell growth. To test this, we compared the content of mitochondria DNA as opposed to genomic DNA. Our result revealed that LKB1 increased the ratio of mitochondria DNA, which was markedly diminished by ablation of the β subunits (Figure 5A). Interestingly, when the ratio of mitochondria protein to total cellular protein was examined, a moderate but non‐significant increase in the ratio was observed in the cells containing LKB1; however, a marked decrease in mitochondrial proteins occurred in the absence of the β subunits, suggesting that the basal level of AMPK activity is required to maintain mitochondria mass (Figure 5B). Furthermore, when the cells were infected with the active mutant of AMPK or GFP as a control, the ratio of mitochondrial to genomic DNA was enhanced to different degrees depending on whether the cells contained β subunits or LKB1(Figure 5C). However, the mitochondrial DNA was not increased when the active mutant of AMPK was targeted to mitochondria (Figure 5D).

**Figure 6:**
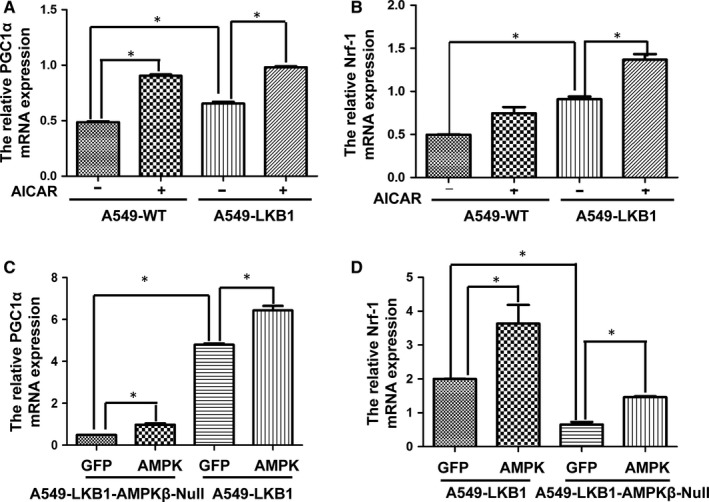
Regulation of PGC1α and Nrf‐1 by AMPK activators. A and B, mRNA levels of PGC1α (A) and Nrf‐1 (B) were measured using qPCR in A549 cells and A549‐LKB1 cells treated with or without ACIAR (1 mmol/L) for 8 h. C and D, mRNA levels of PGC1α (C) and Nrf‐1 (D) were measured in A549‐LKB1 or A549‐LKB1‐AMPKβ null cells infected with adenovirus expressing GFP or active mutant of AMPKα1. Graphs represent means (±SD) (n = 4). Student's *t* test was used to test significance. *P < 0.05

Our next study showed that nuclear transcription factors crucial for the mitochondria biogenesis were modulated by AMPK. Thus, mRNAs for PGC1α and Nrf‐1 were up‐regulated by the presence of LKB1 and/or AICAR (Figure 6A and B). When AMPK β subunits were deleted, mRNAs for PGC1α and Nrf‐1 were down‐regulated (Figure 6C and D). Under this context, the expression of the active mutant of AMPKα1 restored the levels of their mRNAs to some degrees.

### 3.4 AMPK regulates energy metabolism

**Figure 7:**
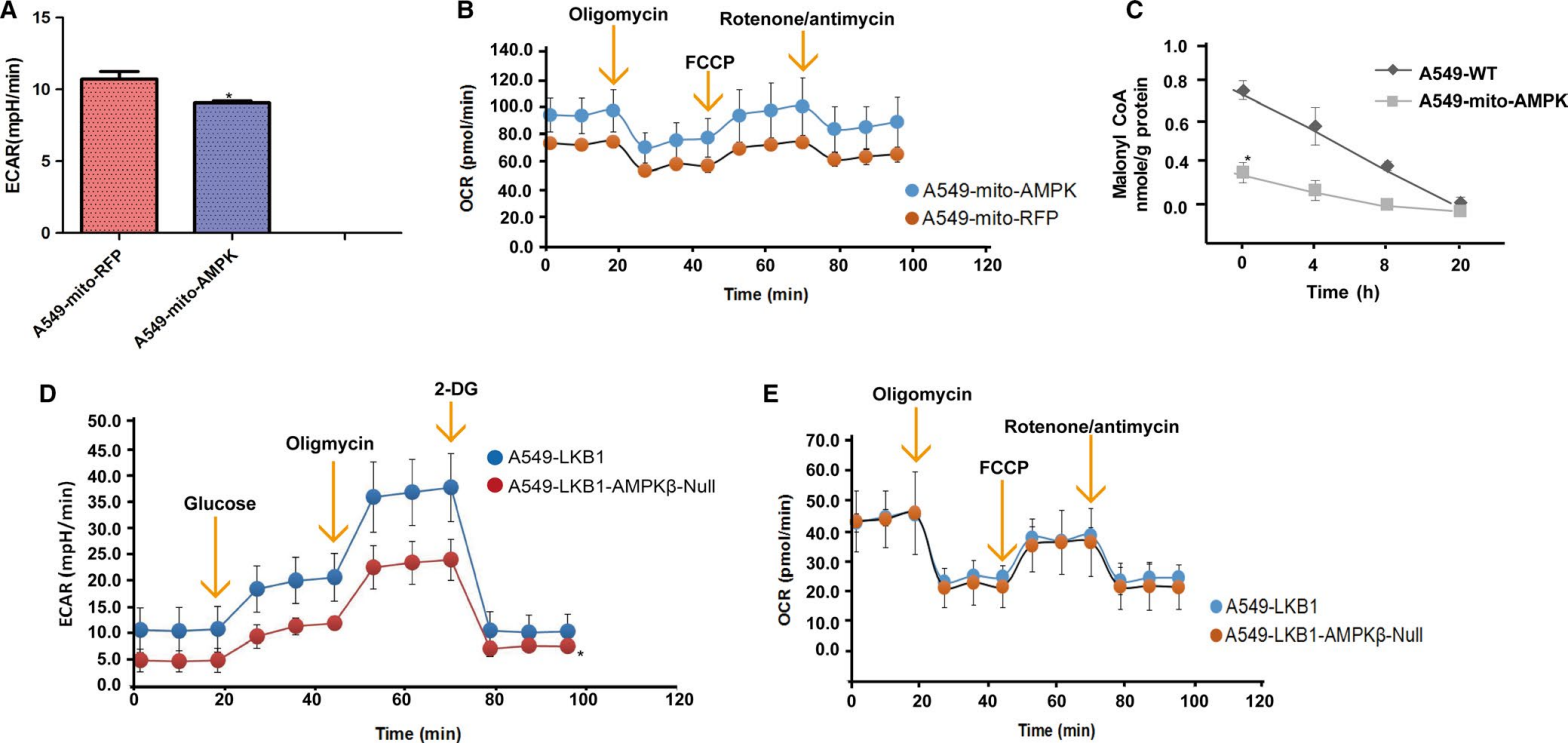
The effects of AMPK on glycolysis and oxidative phosphorylation. Changes in cellular metabolisms were assessed using Seahorse Bioscience XF. A and D, Extracellular acidification rate (ECAR), an indicator of glycolysis rate. B and E, Oxygen consumption rate (OCR), an indicator of oxidative phosphorylation. C, Concentrations of Malonyl CoA were measured from A549 cells and A549‐expressing the active AMPKα1 mutant. Graph represents means (±SD) (n = 5). Two‐way ANOVA was used to test significance. *P < 0.05

Using Seahorse Bioscience XF Analyzer, we carried out extracellular flow analysis to examine the effect of total AMPK and mitochondrial AMPK on mitochondrial respiration and the glycolytic rate. The mitochondrial respiratory rate is reflected by OCR, while extracellular acidification rate (ECAR) measures glycosylation rate. Our results showed that targeting the active mutant of AMPK to mitochondria led to a decrease in basal level of ECAR and an increase in OCR (Figure 7A and B). This was consistent with findings from biochemical measurement of lactic acid in tissue culture medium (data not shown) and assays on cellular levels of malonyl CoA (Figure 7C). The latter is a product of carboxylation of acetyl CoA catalysed by acetyl CoA carboxylase (ACC), which was the first substrate of AMPK identified to be phosphorylated and inhibited. Excessive malonyl CoA inhibits carnitine acyltransferase 1 in the mitochondrial membranes. This enzyme transports long‐chain fatty acyl CoA into mitochondria for β‐oxidation. Thus, targeting AMPK to mitochondria decreased malonyl CoA, leading to increased fatty acid oxidation. We then examined OCR, ECAR and release of lactic acid in the A549‐LKB1 cells with or without ablation of the β subunits. Deletion of the β subunits caused a decrease in ECAR, but no significant change in OCR (Figure 7D and E). Biochemical assays also showed the decrease in lactic acid concentration (data not shown). To our surprise, the overall OCR levels in A549‐LKB1 cells were lower than those in A549 cells. The underlying reason is not known, but it is possibly that this was caused by the fact that reduced proliferation of the cells expressing LKB1 diminished the demand for ATP.

## 4 DISCUSSION

The present study delineated the regulation of mitochondrial functions in cancer cells. On the one hand, we found that AMPKα subunit bound to mitochondria independently of β subunit and that targeting AMPK to mitochondria facilitated fatty acid oxidation and oxidative phosphorylation, and inhibited glycolysis. As a result, mitochondrial AMPK inhibited the growth of cancer cells and tumorigenesis. On the other hand, our study showed that knockout of the β subunits completely abolished AMPK activity, reduced mitochondria DNA and protein contents, which was rescued to some degrees by overexpression of the active mutant of bulky cellular AMPK, but not the mitochondrial counterpart. Consistently, our data revealed that the transcriptional factors PGC1α and Nrf‐1 were regulated by LKB1/AMPK and that the cells lacking the β subunits failed to up‐regulate these two factors in the presence of active mutant of α1 subunit. Finally, we found that lack of total AMPK activity diminished glycolysis, suggesting its requirement for basal energy metabolism. Collectively, our results demonstrate that AMPK is required for regulation of mitochondrial mass through nuclear events and that AMPK regulates oxidative phosphorylation through direct binding to mitochondria. Therefore, we conclude that AMPK exerts integrated effects on mitochondrial function and inhibits tumorigenesis.

Considerable amount of research has demonstrated that AMPK plays negative effects on the growth of cancer cells.^20^, ^27^ It has been shown that AMPK activators including metformin, AICAR, berberine, A769962 and aspirin suppress the growth of cancer cells in vitro and in vivo, which can be mimicked by the active mutant of AMPKα subunit or prevented by its dominant negative mutant. Secondly and more importantly, clinical investigations reported reduced incidence of cancer in type 2 diabetes taking metformin and a preventive effect of aspirin on cancer.^28^, ^29^ Thirdly, previous studies have reported reduced expression of AMPK in breast and other cancer specimens as compared to adjacent normal tissues and complete pathological responses in a clinical trial of metformin as a neoadjuvant chemotherapy.^30^, ^31^, ^32^ Pineda et al^33^ have delineated that MAGE3 and 6, two proteins normally restricted in testis, but reactivated in many human cancers, tether AMPKα1 and TRIM28, an E3 ubiquitin ligase, leading to the degradation of the former. This was believed to be a widespread mechanism of α1 reduction in human cancers. Fourthly, AMPK is situated in the centre of tumour suppressing network of LKB1, TSC2 and p53, all of which are involved in maintaining energy balance, synthesis of macromolecules, cell cycle progression, autophagy and apoptosis.^27^ In fact, deletion of AMPKα1 alleles in mouse accelerates development of Myc‐driven lymphoma and enhances aerobic glycolysis of cancer cells.^25^ In addition, other studies have shown that AMPK activation inhibits Warburg effect and favours oxidative phosphorylation.^25^, ^34^, ^35^ AMPK has been shown to phosphorylate and inhibit MDM2, thereby enhancing p53 tumour suppressive function.^36^ Finally, many studies have reported that AMPK inhibits epithelial to mesenchymal transition and thus prevents cancer cell progression.^22^, ^23^, ^37^ All these studies support that AMPK acts as a tumour suppressor.

In contrast, a number of studies have reported the protecting and promoting effects of AMPK on cancer.^38^, ^39^, ^40^, ^41^, ^42^ How do we reconcile these two paradoxical disparities? The notion on the promoting effect of AMPK on cancer is usually based on the observations under stress settings, while its tumour suppressive function is grounded on the effects of pharmacological activators or AMPK overexpression on bulky tumour. Since AMPK is a stress activated protein kinase and its activation under stress confers protection to cells. During the course of cancer development, prior to build‐up of blood vessels hypoxia and shortage of nutrients exist in cancer microenvironment at some time points or even different sites of cancer at all times. This hypoxic condition leads to activation of AMPK, which induces autophagy to supply nutrients from self‐digestion of macromolecules or cell organelles, so as to copy with energy crisis. Autophagy can only confer protection to cancer cells for certain period of time, but would lead to apoptosis if it exceeds a certain point. If prolonged activation of AMPK is achieved by pharmacological agents or active mutants, the opposite effect could occur.

Mitochondria association of AMPK was recently described by Liang et al.^42^ It was shown that myristoylation of the β subunits targets AMPK to mitochondria and targeting β1 subunit to mitochondria leads to mitophagy and promotes survival of cancer cells. Previous studies from the same laboratory have also demonstrated the inhibitory effect of AMPK, which is linked to two tumour suppressors, p53 and p27.^43^, ^44^ Collectively, the studies from this laboratory seemed to distinguish the functions between mitochondria AMPK and bulky AMPK. Our present study is different from the findings of Liang et al. First, we found that α subunits associated with mitochondria independently of the β subunits. Second, our results showed that targeting the active mutant of α1 subunit to mitochondria led to inhibition of cancer cell growth and also tumorigenesis, supporting a tumour suppressive function.

Previous studies have illustrated important roles of AMPK in functions of mitochondria. AMPK is known to regulate mitochondria biogenesis via regulation of PGC1α in response to exercise, hypoxia and pharmacological activators.^45^, ^46^ Deletion of β1β2 subunits was shown to reduce muscle mitochondria contents.^47^ In addition, AMPK activation increases fission rate and reduces fusion rate of mitochondria, processes associated with mitophagy and apoptosis.^48^ In the present study, we deleted both β1 and β2 subunits and found that mitochondria mass was greatly diminished. Without the β subunits, the expression of α subunits was also reduced which is consistent with a previous report.^49^ It would be plausible to predict that glycolysis would increase in the absence of AMPK activity, if AMPK favoured mitochondrial oxidative phosphorylation. However, to our surprise, our results revealed that cells lacking total AMPK activity exhibited a decrease in glycolysis without a significant compensation of oxidative phosphorylation, as compared to control cells. One explanation is that AMPK regulates extra‐mitochondrial events in glucose catabolism. Indeed, AMPK was reported to increase flux through the glycolysis pathway by phosphorylating 6‐phosphofructo‐2‐kinase/fructose‐2,6‐bisphosphatase 3 (PFKBP3), which via fructose‐2,6 bisphosphate regulates the activity of PFK1, a rate‐limiting enzyme in glycolysis.^50^ It is possible that in the absence of AMPK activity, this step is suppressed, resulting in reduced glucose flux to both glycolysis and mitochondria oxidation.

In conclusion, our current work presents two novel pieces of findings. First, our study bifurcates the function of AMPK in regulation of mitochondria. While cytosolic AMPK regulates mitochondria biogenesis and glucose catabolism, mitochondria‐associated AMPK stimulates oxidative phosphorylation and inhibits aerobic glycolysis. Second, we have shown that mitochondrial AMPK inhibits the growth of cancer cells both in vitro and in vivo. Our next task is to identify specific substrates of AMPK located in mitochondria and elucidate the mechanism underlying tumour suppressive function of mitochondrial AMPK.

## CONFLICT OF INTEREST

We hereby declare that all co‐authors have no conflict of interest in this work, including affiliations, financial relationships, personal relationships or funding sources that could be perceived as influencing an author's objectivity regarding the manuscript content.

## AUTHOR CONTRIBUTIONS

SSJ, YW, LYL, FLS, JRZ, HL, YY, YFL, ZZ and BZ performed the experiments and contributed to data analysis; SSJ, PJL and ZJL contributed to writing of the manuscript; SSJ, DQH and ZJL contributed to experimental design and interpretation of experimental data. All authors gave final approval.
